# Investigating Emotional Top Down Modulation of Ambiguous Faces by Single Pulse TMS on Early Visual Cortices

**DOI:** 10.3389/fnins.2016.00305

**Published:** 2016-06-30

**Authors:** Zachary A. Yaple, Roman Vakhrushev, Jacob Jolij

**Affiliations:** ^1^Centre for Cognition and Decision Making, National Research University Higher School of EconomicsMoscow, Russia; ^2^Department of Experimental Psychology, University of GroningenGroningen, Netherlands; ^3^Department of Psychology, National Research University Higher School of EconomicsMoscow, Russia

**Keywords:** emotion dependent top-down processing, face perception, occipital face area, primary visual cortex, TMS

## Abstract

Top-down processing is a mechanism in which memory, context and expectation are used to perceive stimuli. For this study we investigated how emotion content, induced by music mood, influences perception of happy and sad emoticons. Using single pulse TMS we stimulated right occipital face area (rOFA), primary visual cortex (V1) and vertex while subjects performed a face-detection task and listened to happy and sad music. At baseline, incongruent audio-visual pairings decreased performance, demonstrating dependence of emotion while perceiving ambiguous faces. However, performance of face identification decreased during rOFA stimulation regardless of emotional content. No effects were found between Cz and V1 stimulation. These results suggest that while rOFA is important for processing faces regardless of emotion, V1 stimulation had no effect. Our findings suggest that early visual cortex activity may not integrate emotional auditory information with visual information during emotion top-down modulation of faces.

## Introduction

When perceptual input is ambiguous observers may rely on contextual information in order to process what we see (Jolij and Meurs, 2011). The fact that contextual information can be employed for top-down modulation suggests that perception is rather Bayesian in nature, in that observers generate the most likely interpretation of our visual input relying on contextual information, memories and expectations (Bar, 2004; Kersten et al., 2004; Summerfield and Egner, 2009).

Top-down modulation of perception is not only facilitated by what we expect, but is also contingent on our emotional state. In some studies participants tend to show an emotional bias toward ambiguous stimuli in emotion-related paradigms. For example, when subjects in a negative mood simultaneously view ambiguous faces, participants tend to judge ambiguous facial expressions as sad (Bouhuys et al., 1995; Niedenthal, 2007).

Enhancing emotional significance of stimuli using music has been shown to influence the perception of facial expressions. Emotion laden influences on perception have been demonstrated in a face detection task while subjects listen to happy and sad music (Jolij and Meurs, 2011; Jolij et al., 2011). Jolij and Meurs (2011) were able to demonstrate that when subjects rated facial features of emoticons as happy or sad while passively listening to happy or sad music, participants became more sensitive to emoticons that were congruent with the music mood. They explained this phenomenon by suggesting that perception may be influenced by emotional state in a top-down manner.

For this study we plan to investigate the influence of emotional state on perception of ambiguous faces within the visual cortex. While some studies have been conducted on top-down emotional influence of perception of faces (Baumgartner et al., 2006; Li et al., 2009; Jeong et al., 2011; Müller et al., 2012), no studies have investigated the role of the early visual cortex in the context of emotional top-down perception of faces. In particular, we will investigate the role of the rOFA, a region within the inferior occipital gyrus which processes face parts (Rossion et al., 2003; Pitcher et al., 2007; Liu et al., 2010; Nichols et al., 2010; Renzi et al., 2015), has been linked with integration of facial stimuli (Cohen Kadosh et al., 2011), and becomes activate 60–100 ms after stimulus onset, prior to the fusiform gyrus (Pizzagali et al., 1999; Halgren et al., 2000; Liu et al., 2002; Rossion et al., 2003; Pitcher et al., 2007, 2008; Rotshtein et al., 2007; Cohen Kadosh et al., 2010; Sadeh et al., 2010; Pitcher, 2014). In addition to the rOFA, we chose to stimulate V1 since it has been shown to be relevant for auditory-visual integration (Clavagnier et al., 2004; Muckli et al., 2015, and becomes activate during face perception—around the same time frame as the rOFA, ~90 ms (Pourtois et al., 2004).

The purpose of the study is to investigate the role of the early visual cortex during emotion dependent top-down processing of faces. To this end, we will use single pulse TMS, to disrupt activation of rOFA and V1 while subjects identify ambiguous facial expressions and simultaneously listen to happy and sad classical music. We expect that stimulation of the rOFA and V1 will interfere with identification of happy and sad ambiguous faces, especially when music and faces are mismatched in emotional content.

## Methods and materials

### Subjects

Twenty-four right-handed Bachelor students (15 males, mean age 22 years, SD 1.4 years) at the University of Groningen with normal or corrected-to-normal vision were recruited for the study. Each recruit was paid 7 euros per hour for the entire 2.5 h they had spent in the lab. Participants either taking drugs or prescribed with medications were excluded from the participant pool. The study was permitted by the local Ethics Committee (“Ethische Commissie van het Heymans Instituut voor Psychologisch Onderzoek”) and conducted according to the Declaration of Helsinki. Written informed consent was acquired from all participants.

### Visual stimulation

**Figure 1:**
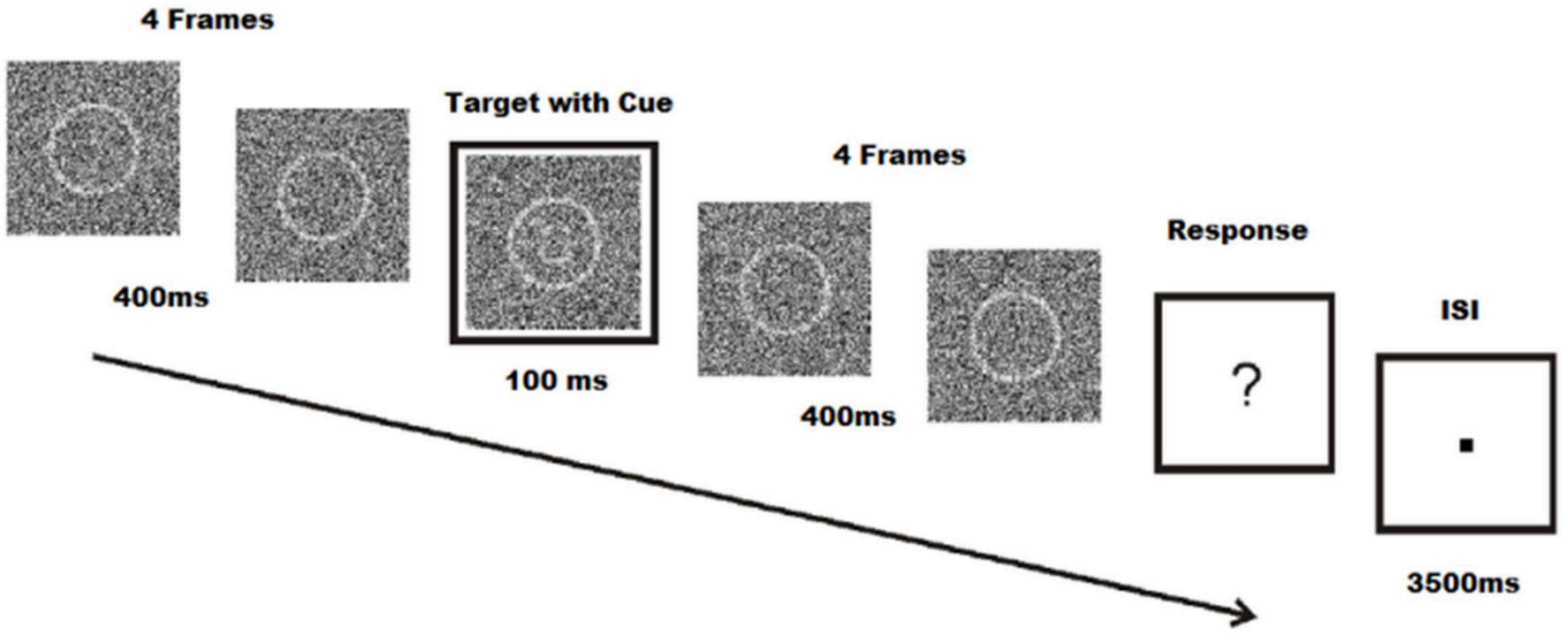
**Face-detection task**. Participations identify facial expressions of emoticons during a 100 ms target frame with cue. Each target, coupled with four pre- and post-frames are masked with noise. Subjects are required to respond even when no face is seen. Modified from Jolij and Meurs (2011).

Stimuli were created using Matlab R2010a (The Mathworks Inc., Natick, Massachusetts, USA), and consisted of a 140 by 140 pixels array of random noise. Within the random noise, a circle with a diameter of 74 pixels was centered and shaded with a lighter contrast. All 255 values of the grayscale palette were used to create the random noise images. The difference between target and non-target stimuli was that the target stimuli also contained eyes, eyebrows, and a mouth within the circle, forming a graphic representation of a face with either a sad or a happy expression. Schematic faces have been shown to have similar emotional content compared to real faces, and may be used to influence emotion in perceptual tasks (Jolij and Lamme, 2005). Stimuli were presented on a 190 Philips Brilliance 190B TFT monitor (Philips BV, Eindhoven, The Netherlands) with a resolution of 800 by 600 pixels; viewing distance was ~80 cm. Trials were animations of 9 frames, each frame lasting 100 ms. The fifth frame also included a cue, a white square around the noise patch which also lasts for 100 ms. After the 9 frames, subjects were presented with a fixation dot until they responded. Once they responded, subjects were presented with an inter-stimulus interval between 3500 and 4500 ms. The following trial began after the inter-stimulus interval (Figure 1). Target and non-target stimuli were chosen at random in each trial to avoid expectancy or learning effects. Noise patches were randomly chosen from a list of 500 different noise patches. Within these noise patches, targets were chosen from a series of 50 happy and 50 sad images. While schematic faces were identical, noise patches added to the schematic face stimuli varied the stimuli.

### Music manipulation

While performing the task participants listened to classical music which lasted 30 min maximum for all blocks in the happy and sad music condition. For the happy music blocks, pieces that had higher and more frequent pitches (faster beats per minute) as well as a dominant major key were chosen to represent happy music. Conversely, pieces in the key of a minor chord and those with a slower tempo were chosen to represent sad emotional music. Songs were played in random order. Music type was counterbalanced across subjects. Music was played via an MP3-player with a handheld speaker. Headphones were not used because the TMS stimulation would cause interference when stimulating the rOFA. Participants chose a volume that they felt comfortable with. Happy music pieces: Violin Concerto In A, Rv 347 (Ed. Malipiero)—I Allegro; Vivaldi—Concerto for Guitar and String Orchestra in A Major—I. Allegro non-molto; Concerto No. 1 In E, Rv 269 “spring”—I Allegro; Vivaldi—Concerto for Guitar and String Orchestra in A Major—III. Allegro; Vivaldi—Concerto for Guitar and String Orchestra in D major—III. Allegro; Concerto No. 1 In E, Rv 269 “spring”—Iii Allegro; Concerto No. 3 In F, Rv 293 “autumn”—Iii Allegro; Violin Concerto In A, Rv 347 (Ed. Malipiero)—Iii Allegro. Sad music pieces: Beethoven Moonlight Sonata (i), Chopin Nocturne No. 20 in c# (The Pianist), Chopin Prelude 6, Chopin Etude 6 Chopin Prelude No. 20 in c minor, Violin Concerto In A Minor, Rv 356 (Ed. Malipiero)—Ii Largo, Scriabin Etude Op2 No1 (1887), Barber Adagio for Strings see Supplementary Material for full list of music pieces.

### Mood assessment

Participants' emotional state was evaluated by using the Self-Assessment Manakin (SAM), a non-verbal method used to assess the valence, arousal, and dominance associated with a person's emotional state (Bradley and Lang, 1994). For this experiment, the SAM is utilized to assess the contrast in arousal and emotional valence between music and no music conditions while performing the task. Previous research has been done using the SAM to assess mood while performing a face-detection task and listening to music (Jolij and Meurs, 2011). The authors demonstrated that while music increases arousal level, music mood may influence subjects' emotional valence depending on the direction of the music mood.

### Experimental procedure

Subjects are randomly assigned to one of three TMS locations (rOFA, V1 and Cz as a baseline stimulation). Subjects were given a total of 4 blocks for each music condition (happy, sad, no music) equating to 12 blocks in total. Each block consisted of 72 trials, divided into happy, sad or no face trials. TMS pulses were fixed at 80 ms after stimulus onset for all conditions. We settled with a fixed stimulation time because our main hypothesis concerned the role of the rOFA which activates at 80 ms. Participants had to indicate whether they had seen a sad face or a happy face by using the computer keyboard (the F and J keys, respectively), or to press the Spacebar when they had not seen a face. Participants were specifically instructed to be conservative with their responses, i.e., to only respond when they were absolutely sure to have seen a face. Trials were discarded from the average reaction times if they responded later than 3 s after stimuli onset. No feedback was given. Participants' mood was assessed using the SAM immediately after each music condition.

### Magnetic stimulation and coil positioning

The TMS regions of interest were measured by first converting foci of the rOFA (Rossion et al., 2003) into the EEG 10–20 system using T2T Munster (http://archive.is/wwwneuro03.uni-muenster.de). Cz was located by marking 50% between the inion to nasion and 50% between the left and right preauricular points. V1 was located at electrode Oz, 10% above the inion. The rOFA was located at electrode T6 by scoring 10% above the inion (Oz), 10% above the right periauricular point (T4) and 20% of the total circumference between Oz and T4, ~5 cm to the right of V1. Stimulation of TMS was applied using a Magstim Model 200 stimulator (The Magstim Company Ltd, Wales, UK), using a 70 mm figure-of-eight coil. Intensity was set for below visual threshold for each subject (between 65 and 80% for all subjects).

### Statistical analyses and *post-hoc* comparisons

Data were analyzed using SPSS version 18.0.03 (SPSS Inc., Natick, Massachucetts, USA). Mixed ANOVA tests were performed on accuracy and reaction time of facial expression identification (correct detection of face) for a within subject's factor: *Congruence* (No music, Congruent, Incongruent) and between subject's factor: *Location* (rOFA, V1, Cz). A separate mixed ANOVA was performed to test accuracy on arousal of music: *Music condition* (No music, Sad, Happy), *Face condition* and between subject's factor: *Location* (rOFA, V1, Cz). *Post-hoc* comparisons were corrected using Bonferonni procedure. Sphericity was not violated for any of the effects (all *p* > 0.05). *T*-tests were used to analyze three scales of the SAM (valence, dominance, and arousal) for each music condition.

## Results

### SAM questionnaire

The valance scale of the SAM revealed a main effect of music mood suggesting that subjects report a more positive mood after listening to happy music and report a more negative mood after listening to sad music (*F* = 23.995, *p* < 0.01). The arousal scale of the SAM revealed higher arousal when listening to sad music compared to happy and no music condition (*F* = 8.556, *p* = 0.002). No differences in the dominance scale were tested.

### Behavioral data

The mixed ANOVA on *Congruency* × *Location* revealed a two-way interaction effect [*F*_(4, 42)_ = 2.993, *p* = 0.031, partial η^2^ = 0.219]. When comparing across *Congruency*, differences were observed between congruent and incongruent for subjects that received stimulation on Cz (*p* = 0.043).

When comparing across *Location*, Bonferroni corrected comparisons revealed statistical significance in accuracy between groups receiving rOFA and Cz stimulation for congruent face/music stimuli (*p* < 0.001) and stimuli with no music (*p* = 0.005). Statistical differences were also found between rOFA and V1 for congruent stimuli (*p* < 0.001) and the no music condition (*p* = 0.009). Both of these effects were driven by a decrease in performance rOFA stimulation.

**Figure 2:**
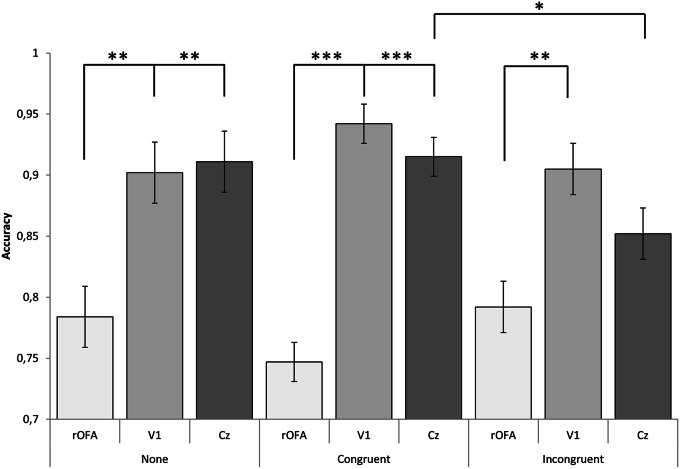
**Graphical representation of interaction effect**. An effect between *Congruence* and *Location* reflects emotion dependent top-down influence of face perception at baseline level (Cz), yet for V1 and rOFA, no effects were found. Instead, rOFA disrupted perception of faces regardless of emotion content while V1 stimulation facilitated performance. ^***^p < 0.001; ^**^p < 0.01; ^*^p < 0.05; *p* < 0.10.

Conversely, differences between rOFA and Cz groups were non-significant for incongruent stimuli (*p* = 0.186) and different between rOFA and V1 stimulation (*p* = 0.004). No differences were observed between V1 and Cz across *Congruency*, see Figure 2.

Results also revealed a main effect of location [*F*_(2, 21)_ = 25.467, *p* < 0.001, partial η^2^ = 0.708]. Subjects receiving rOFA stimulation correctly identified 77% of faces, ~14% less than subjects receiving V1 stimulation (μ = 92%; *p* < 0.001) and 12% less than subjects receiving Cz stimulation (μ = 89%; *p* < 0.001).

Furthermore, no effects were found significant for reaction time across conditions. Finally, due to an increase in arousal level for sad music compared to the other two conditions, we divided *Congruency condition* into *Music condition* and *Face condition* and compared behavioral scores on face identification. This analysis revealed no effects on *Music condition* alone, indicating that the effects reported above were merely due to incongruent music and face stimuli.

## Discussion

For this study we investigated the functional role of early visual cortical areas involved in emotion dependent top-down modulation on the perception of facial expressions. This was done by stimulating rOFA and V1 while subjects identified ambiguous happy and sad faces and simultaneously listened to happy and sad music. Similarly to a previous behavioral study, performance of face identification during conditions of incongruent music mood decreased with respect to conditions of congruent music mood, yet only at baseline level. During stimulation of the rOFA and V1, identification of faces was modulated, irrespective of congruence between music mood and faces. While rOFA *decreased* performance of face identification in all music-face pairings, V1 increased performance across conditions.

In contrast to our expectations, rOFA stimulation decreased face perception, irrespective of auditory stimuli. The finding that music did not facilitate or impede facial expression identification compared to no music condition demonstrates how the rOFA is selective to face processing. One potential explanation may be that stimulation time was fixed at 80 ms for all conditions. Since this time is critical for rOFA during face processing (Pitcher, 2014), this may explain how performance of face identification decreased equivalently across music mood. On the other hand, V1 did not manipulate performance of face identification compared to baseline, which was not according to our expectations. Although V1 becomes activate early on, the role of V1 may not be specific to emotional dependent top-down modulation on face perception. It has been shown, however, that multisensory integration occurs from long-distance feedback connections projecting from higher cortical areas to V1 in a top-down hierarchical fashion (Ahissar and Hochstein, 2000; Juan and Walsh, 2003; Clavagnier et al., 2004; Muckli et al., 2015). This may suggest that the V1 becomes active for emotional dependent top-down modulation at a later duration, after activation of higher cortical areas.

Compared with previous studies, incongruent music-face pairings shows activation of the bilateral fusiform gyrus (Jeong et al., 2011), while congruent auditory-visual stimuli activates the right fusiform gyrus (Baumgartner et al., 2006). However, in this latter study classical music and fearful and sad pictures was used to induce emotional congruence, rather than face stimuli. This may suggest that while the right fusiform gyrus may account for auditory-visual integration of emotional stimuli in general, the rOFA specifically may account for emotional auditory-visual integration of faces. Further research is necessary to explore the distinctive functional roles of rOFA and right fusiform gyrus in face perception while influenced by emotional state.

In this study we stimulated early visual cortical regions to investigate emotion dependent top-down processing of faces. However, perhaps the influences of face perception may be attributed to higher cortical areas. For example, the superior temporal region has been associated with the binding of faces with voices (Watson et al., 2013, 2014a,b). In particular, the posterior superior temporal sulcus plays a crucial role in audio-visual integration which gates influences from the fusiform gyrus toward the left amygdala when processing emotion-laden faces (Müller et al., 2012). Furthermore, generation of top-down signals during face perception revealed activity within the anterior cingulate cortex, orbitofrontal cortex and left dorsolateral prefrontal cortex that (Li et al., 2009). Therefore, emotional state may influence face perception from higher cortical regions such as the superior temporal cortex, anterior cingulate cortex, orbitofrontal cortex or left dorsolateral prefrontal cortex. For this reason, it is necessary to further explore neural networks involved in emotion dependent top-down modulation in order to understand the how emotional state influences visual perception of faces.

## Conclusion

Earlier studies suggest that V1 facilitates integration of multisensory stimuli while the rOFA is involved in processing of face parts. For this study we attempted to investigate the role of the rOFA and V1 during emotion dependent top-down modulation of face perception. Subjects passively listened to emotionally congruent or incongruent music were instructed to identify ambiguous happy and sad faces in a face-detection task. Results provided evidence of emotional influence on face perception in the control (Cz) stimulation condition, yet stimulation of rOFA decreased accuracy of face identification, irrespective of congruency. No effect was found between V1 and baseline. These results suggest that while the rOFA has more general role of face perception, early activation of V1 (80 ms) did not manipulate emotion dependent top-down modulation.

## Author contributions

Data collection, analysis, and writing of report was mainly performed by ZY. RV assisted analysis and writing of report. Study design and supervision was conducted by JJ.

### Conflict of interest statement

The authors declare that the research was conducted in the absence of any commercial or financial relationships that could be construed as a potential conflict of interest.
