# Corrigendum: Investigating Emotional Top Down Modulation of Ambiguous Faces by Single Pulse TMS on Early Visual Cortices

**DOI:** 10.3389/fnins.2016.00501

**Published:** 2016-11-10

**Authors:** Zachary A. Yaple, Roman Vakhrushev, Jacob Jolij

**Affiliations:** ^1^Centre for Cognition and Decision Making, National Research University Higher School of EconomicsMoscow, Russia; ^2^Department of Experimental Psychology, University of GroningenGroningen, Netherlands; ^3^Department of Psychology, National Research University Higher School of EconomicsMoscow, Russia

**Keywords:** emotion dependent top-down processing, face perception, occipital face area, primary visual cortex, TMS

Due to an oversight, the original article did not include the supervisor of this thesis project, Dr. Jacob Jolij, who substantially contributed to the research project. The updated author list appears above while the updated Author Contributions statement and Acknowledgments appear below.

The authors apologize for this error. This does not affect the scientific conclusions of this article in any way.

The original article has been updated.

## Author contributions

Data collection, analysis, and writing of report was mainly performed by ZY. RV assisted analysis and writing of report. Study design and supervision was conducted by JJ.

### Conflict of interest statement

The authors declare that the research was conducted in the absence of any commercial or financial relationships that could be construed as a potential conflict of interest.

